# Field scale biodegradation of total petroleum hydrocarbons and soil restoration by Ecopiles: microbiological analysis of the process

**DOI:** 10.3389/fmicb.2023.1158130

**Published:** 2023-04-21

**Authors:** Ruben Martínez-Cuesta, Robert Conlon, Mutian Wang, Esther Blanco-Romero, David Durán, Miguel Redondo-Nieto, David Dowling, Daniel Garrido-Sanz, Marta Martin, Kieran Germaine, Rafael Rivilla

**Affiliations:** ^1^Departamento de Biología, Universidad Autónoma de Madrid, Madrid, Spain; ^2^EnviroCore, Dargan Research Centre, South East Technological University, Carlow, Ireland

**Keywords:** Ecopile, hydrocarbon, bioremediation, microbial succession, microbiota

## Abstract

Ecopiling is a method for biodegradation of hydrocarbons in soils. It derives from Biopiles, but phytoremediation is added to biostimulation with nitrogen fertilization and bioaugmentation with local bacteria. We have constructed seven Ecopiles with soil heavily polluted with hydrocarbons in Carlow (Ireland). The aim of the study was to analyze changes in the microbial community during ecopiling. In the course of 18 months of remediation, total petroleum hydrocarbons values decreased in 99 and 88% on average for aliphatics and aromatics, respectively, indicating a successful biodegradation. Community analysis showed that bacterial alfa diversity (Shannon Index), increased with the degradation of hydrocarbons, starting at an average value of 7.59 and ending at an average value of 9.38. Beta-diversity analysis, was performed using Bray-Curtis distances and PCoA ordination, where the two first principal components (PCs) explain the 17 and 14% of the observed variance, respectively. The results show that samples tend to cluster by sampling time instead of by Ecopile. This pattern is supported by the hierarchical clustering analysis, where most samples from the same timepoint clustered together. We used DSeq2 to determine the differential abundance of bacterial populations in Ecopiles at the beginning and the end of the treatment. While TPHs degraders are more abundant at the start of the experiment, these populations are substituted by bacterial populations typical of clean soils by the end of the biodegradation process. Similar results are found for the fungal community, indicating that the microbial community follows a succession along the process. This succession starts with a TPH degraders or tolerant enriched community, and finish with a microbial community typical of clean soils.

## 1. Introduction

The biodiversity of the soils is often threatened by contamination, which is a collateral effect of industry, mining, and agriculture and represents a great concern from an economic, environmental, and social standpoint (Gardi et al., 2013). Soil pollutants can be classified into organic or inorganic depending on their chemical composition. Organic pollutants include, among others, derivatives of petroleum products and are divided into two main groups: aliphatics and aromatics (Mostert et al., 2010). Aliphatic compounds present in petroleum hydrocarbon products are mostly represented by alkanes of straight or branched-chains (Militon et al., 2010; Khan et al., 2018). Aromatic compounds range from those composed of one benzene ring to complex chemicals formed by multiple fused rings and include polycyclic aromatic hydrocarbons (PAHs) (Khan et al., 2018). PAHs are considered persistent organic pollutants due to their resistance to being degraded through chemical or biological processes (Ying and Wei, 2019). They can be released by incomplete combustion of organic materials, and are known carcinogenic, genotoxic, and mutagenic compounds (Pinedo et al., 2013; Das, 2014; Crampon et al., 2018; Khan et al., 2018). Total Petroleum Hydrocarbons (TPHs) are mainly composed by aliphatic and aromatic fractions (Khan et al., 2018). They are commonly released into the environment by accidental spills and leaks during their transport or storage. When these leaks occur, TPHs are usually placed in the outer layers of the soils (Varjani, 2017), where they alter the physical properties such as pH, bioavailability of nutrients or biodiversity (Varjani, 2017; Devatha et al., 2019). Organic pollutants such as TPHs can be biodegraded *via* microbial metabolism, whose pathways can lead to their mineralization (Das and Chandran, 2011; Sun et al., 2015).

Bacteria are known to degrade certain petroleum hydrocarbons. For example, *Acinetobacter* and *Alcanivorax* can degrade short and long-chain alkanes (Rojo, 2009; Wang and Shao, 2014; Obieze et al., 2021). *Sphingomonas*, *Nocardia*, or *Rhodococcus* are known to bioremediate anthracene. Some *Corynebacterium*, *Mycobacterium,* and *Pseudomonas* strains are capable of degrading naphthalene (Mrozik et al., 2003; Gałązka et al., 2018). Fluoranthene can be degraded by *Alcaligenes* and *Mycobacterium* (Mrozik et al., 2003). Moreover, *Azoarcus*, *Ochrobactrum,* or *Burkholderia* can bioremediate various benzene derivatives during denitrification (Gałązka et al., 2018). The metabolism toward pollutants displayed by these and other bacteria can be exploited for the restoration of polluted soils or water bodies. Bioremediation is defined as “the application of the metabolic potential of microorganisms and plants to degrade, transform, or accumulate toxic compounds” (Das, 2014). Bioremediation can suppress the toxic effects of the pollutants by their transformation into less toxic substances or mineralization (Pandey et al., 2009). Bioremediation technologies can be classified into two main approaches depending on the place where they are applied: *in situ* and *ex situ*. The former targets pollutant removal or attenuation at the same place where the pollution occurred. Conversely, *ex situ* techniques are based on the extraction and transport of the contaminated entity to another place for its treatment. Both *in situ* and *ex situ* approaches can be carried out together with other bioremediation technologies. In particular, *ex situ* approaches are often combined with bioaugmentation, when the pollutant-degrading microorganisms are previously grown and added to carry out the biodegradation process, and with biostimulation, which involves the addition of nutrients and supplements, especially oxygen, nitrogen, and phosphorus into the soil, or modifying the pH or temperature to increase the rates of biodegradation carried out by the native microorganisms (Lin et al., 2010; Tyagi et al., 2011). Both bacterial consortia or isolated strains can be used to implement a bioaugmentation approach (Guirado et al., 2021; Garrido-Sanz et al., 2022), of which the former is thought to be more effective, as it contains a wider variety of bacteria, where very few are specific for a particular contaminant and can act synergically (Varjani, 2017; Garrido-Sanz et al., 2018; Auti et al., 2019; Garrido-Sanz et al., 2019).

Ecopiling is an *ex-situ* bioremediation method that involves biopiling, biostimulation, bioaugmentation, and phytoremediation to remove pollutants from soils and sediments (Germaine et al., 2015; Liu et al., 2016; Wang et al., 2021). An Ecopiling approach was designed for the bioremediation of a polluted soil at Carlow, Ireland (Wang et al., 2021). Seven Ecopiles were installed in May 2019 to amend 13,000 tons of polluted soil from a former industrial site that underwent a process of hydrocarbon pollution due to the activity of a sugar-beet refinery plant over 80 years (1926–2005). A diagram showing the structure of an Ecopile is shown in Supplementary Figure S1. Ecopiles were planted with a mixture of *Lolium perenne* (Rye grass) and *Trifolium repens* (White clover). Ecopiles were inoculated with an average 1.1228 l/m^3^ of bacterial consortia (10^6^–10^7^ CFU/ml) that had been previously isolated from the polluted soil, and then cultured, of which *Pseudomonas*, *Extensimonas*, *Fulvimonas*, and *Acinetobacter* were the dominant genera. The TPH contamination levels, as well as the initial microbial community of this soil and its metagenome, have been previously reported (Wang et al., 2021). *Proteobacteria* and *Actinobacteria* dominated the bacterial community and *Lysobacter* was the most abundant genus. The amount of hydrocarbons present in this polluted soil was also previously reported (Wang et al., 2021). In this study, we follow and analyze the bacterial succession taking place in these Ecopiles as well as the evolution of fungal populations as indicators of soil restoration, together with the monitoring of aliphatic and aromatic hydrocarbons.

## 2. Materials and methods

### 2.1. Total DNA extraction and quantification

Soil samples from the seven Ecopiles were collected at five different timepoints: July 2019 (T1) December 2019 (T2), February 2020 (T3), June 2020 (T4), and November of 2020 (T5). T0 corresponds to the building of the Ecopiles in May 2019 and their analysis has been previously shown (Wang et al., 2021). All samples were sieved with a 2 mm net followed by manual homogenization. T2 and T4 samples were used for hydrocarbon (TPH) determination and bacterial community analysis. T1 and T3 samples were used for fungal community analysis. The starting point (T0) and the final time (T5) samples were used to determine hydrocarbon concentrations and fungal and bacterial communities. With this schedule, data for each parameter were obtained with a minimum frequency of every 6 months and data for all parameters were obtained at the start and end timepoints.

Two grams of sample were taken from each Ecopile and the bacteria within sample were resuspended in a saline solution (5 ml of sterile NaCl 0.85%) by vigorous shaking at 2000 rpm for 30 min in a Multi Reax agitator (Heidolph). The liquid phase was centrifuged at 300 g for 30 s to pellet the soil particles and subsequently the supernatant was centrifuged at 8000 g for 10 min to pellet the microorganisms. This pellet was finally resuspended in 1 ml of PBS (phosphate-buffered saline; 137 mM NaCl, 2.7 mM KCl, 10 mM Na_2_HPO_4_, and 1.8 mM KH_2_PO_4_). Sixty microliter of lysozyme (1 mg/ml) were added to each sample, followed by an incubation at 37°C for 1 h. Soil samples of 1 gram were used directly for fungal DNA extraction. In both cases, isolation of total DNA from each sample was carried out in triplicate using the FastDNA Spin Kit for Soil (MP Biomedicals, USA) according to manufacturer indications. The isolated DNA was quantified using NanoDrop spectrophotometer 2000c (Thermo Fisher Scientific) and Qubit 4 fluorometer (Invitrogen).

### 2.2. 16S and 18S rRNA sequencing

For bacterial 16S profiling, the isolated DNA was sent to the Genomic Services at Parque Científico de Madrid (Spain) to sequence the V3-V4 16S rRNA amplicons using the primers: 341F (5′-CCT ACG GGN GGC WGC AG-3′) and 805R (5′-GAC TAC HVG GGT ATC TAA TCC-3′) (Herlemann et al., 2011). Briefly, libraries were prepared with Illumina MiSeq v3 reagent kit according to suppliers’ specifications and sequenced by Illumina MiSeq System to get 2 × 300 bp reads.

Fungal samples were sent to Novogene to sequence the v4-v5 18S rRNA region using as primers 528F (5’-GCG GTA ATT CCA GCT CCA A-3′) and 706R (5′-AAT CCR AGA ATT TCA CCT CT-3′) (Cheung et al., 2010). Libraries were constructed by Novogene specifications and sequenced by Illumina NovaSeq 6,000 system to get 2 × 250 bp reads.

### 2.3. Determination of microbial composition profiles and study of diversity among samples

As a first step, the regions corresponding to the oligonucleotides used in the amplification as well as traces of adapters that might remain in the raw reads were removed using Cutadapt (Martin, 2011). Next, the R package DADA2 v1.18 (Callahan et al., 2016) was used to determine the ASVs in each sample as well as their abundance per sample. ASVs information, nucleotide sequence, and abundance were exported into QIIME2 v2-2021.2 (Bolyen et al., 2019) for further analyses. Within QIIME2 environment, ASVs were aligned using MAFFT (Katoh et al., 2002) to construct a rooted phylogenetic tree with fasttree2 (Price et al., 2010). Taxonomic assignation was carried out with the q2-feature-classifier (Bokulich et al., 2018) based on SILVA 99% 16S and 18S sequence database release 138 (Quast et al., 2013). The SILVA database was processed by trimming the sequences to encompass the amplicons used in this study. From the processed database, a naive Bayes classifier (Murphy, 2006) was constructed and the taxonomic assignment of ASVs was performed using the QIIME2 classify-sklearn tool (Pedregosa et al., 2011).

Alpha- and beta-diversity in bacterial samples were analyzed using the QIIME2 diversity plugin (alpha-rarefaction, core-metrics-phylogenetic and alpha-group significance). The diversity within each sample or alpha-diversity was evaluated for evenness and richness using the Shannon index. The significance of the replicate effect on the samples’ alpha-diversity values was assessed using the Kruskal-Wallis and Wilcoxon signed-rank tests. The beta-diversity was estimated by calculating Bray-Curtis distances (Beals, 1984) among samples. Graphical representations of the diversity analyses were performed in R by importing the QIIME2 output files using the qiime2R package (Bisanz, 2018) so that they could be studied using the Phyloseq package (McMurdie and Holmes, 2013). The Bray-Curtis distance matrix was used to represent PCoA plots and hierarchical clustering using the R packages ggplot2 (Wickham, 2011) and pheatmap (Kolde and Kolde, 2015).

Diversity profiling in fungal samples was similarly studied using Phyloseq and Vegan (Oksanen et al., 2013) packages in R. Initially, the table of taxonomic assignments was refined to replace the taxonomic levels with no result by the Uncl string plus the information of a higher taxonomic level. The same was done for those levels with the string Incertae sedis. To normalize the samples, a rarefaction was performed to obtain a new sample table with a depth equivalent to 90% of the sample with the lowest number of sequences. Subsequently, Shannon indexes and the Bray-Curtis distance table were determined for both individual and merged samples according to sampling time. In addition, the DESeq2 package (Love et al., 2014) was used to compare the differential abundance of populations between the first and last sampling time, selecting those ASVs with a log2FoldChange greater than 2.5 or less than −2.5 for bacteria and 1 and −1 in the case of fungal samples and a p-adjusted value equal to or less than 0.01.

The Vegan R package (Oksanen et al., 2013) was used to perform a non-metric multidimensional scaling (NMDS) analysis to explain the differences between sampling times based on bacterial populations and concentrations of pollutants (explanatory variables), using Bray-Curtis distance transformation and a *k* = 2.

### 2.4. TPHs determination

Samples of soil were analyzed for TPH and polycyclic aromatic hydrocarbon (PAH) concentration. Analysis and speciation were carried out by an accredited external commercial laboratory (ALS Environmental, United Kingdom). TPH extraction was carried out on 10 g of soil sample using a hexane:acetone (50:50) solvent, and the extracts were analyzed using gas chromatography-flame ionization detection (GCxGC-FID). For PAH speciation, 5 g of soil samples was subjected to microwave extraction using a hexane:acetone:trimethylamine (50:45:5) solvent mixture and analyzed by GC-mass spectroscopy (GC–MS).

## 3. Results

### 3.1. Biodegradation of TPHs in Ecopiles

The evolution of the pollutants concentrations in the Ecopiles is shown in Table 1. In May 2019 (T0), when Ecopiles were built, average total hydrocarbons concentration was 9,244 mg/kg of soil, where total aliphatics represented 3,944 mg/kg and total aromatics were 5,350 mg/kg (Wang et al., 2021). After 18 months, at the end of the process (T5), TPHs average was reduced to 686 mg/kg, where 648 mg/kg corresponded to aromatics and only 38 mg/kg to aliphatics. This represents the degradation of 93% of TPHs, with a 99% degradation of aliphatics and 88% depletion of aromatics. These results show that Ecopiles were successful in the degradation of TPHs and that most of the biodegradation was achieved during the first 12 months. Regarding the individual Ecopiles (Supplementary Table S1). Ecopiles 6 and 7 showed the highest concentrations both for aliphatic and aromatic concentrations along the entire bioremediation project. At the end of the bioremediation process, Ecopiles 2 and 4 had the lowest hydrocarbons concentration for both total aliphatics and aromatics (26 and 25 mg/kg for the aliphatics, respectively and 315 and 566 mg/kg for the aromatics, respectively). At this final time, Ecopiles 1 and 7 were the most polluted for aliphatics (66.5 and 53.5 mg/kg, respectively), while 6 and 7 remained as the most polluted for total aromatics (854 and 924.5 mg/kg). Nevertheless, a reduction in aliphatics higher than 98% and in aromatics higher than 82%, was achieved for all the Ecopiles.

**Table 1:** Mean, standard deviation, and percentage against the initial quantity of the petroleum hydrocarbons present in the Ecopiles along the bioremediation process.

Timepoints	Petroleum hydrocarbon fractions (mg/kg)					
	Mean aliphatics	St. Dev aliphatics	% of aliphatics	Mean of aromatics	St. Dev of aromatics	% of aromatics
May 2019 (T0)	3944.4	3300.8	100	5350.3	4435.1	100
December 2019 (T2)	938.1	538.1	23.8	1088.1	800.6	20.3
June 2020 (T4)	32.8	35.3	0.8	241.1	91.2	4.5
November 2020 (T5)	38.4	14.5	1	648.7	185.9	12.1

### 3.2. Evolution of the bacterial community in Ecopiles along the bioremediation process

**Figure 1:**
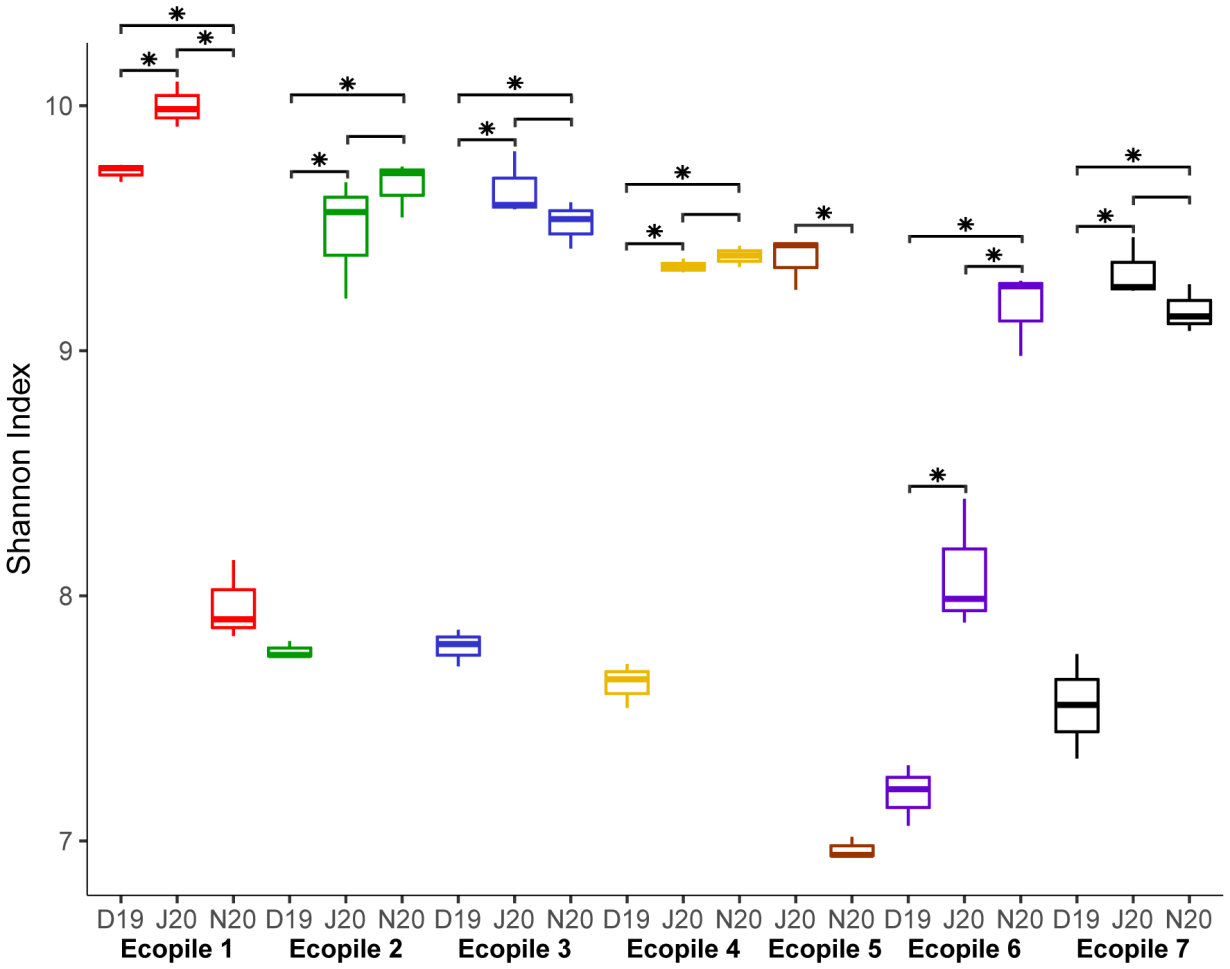
Boxplot representing the bacterial Shannon index values for each timepoint and Ecopile. Boxplots contain the Shannon index values of the three replicates of each sample. Differences between Shannon index values at the different timepoints were assessed with the Kruskal-Wallis test. Asterisks represent the significance at a *p* adjusted-value ≤0.05. Colors of boxplots according to the different Ecopiles.

16S rRNA amplicon sequencing of the seven Ecopiles in the three timepoints (T2, T4 and T5), resulted in an average of 138,221 reads per replicate. After processing, an average of 82,250 high-quality reads per replicate remained, representing 59.5% of the raw reads. The rarefaction curves of all the samples (Supplementary Figure S2) show that a complete coverage of ASVs was achieved, as they reach an asymptote with less than 10,000 sampled sequences. The number of observed ASVs ranged from 1,369 to 3,000 (Ecopiles 6 and 1, respectively) in December 2019, 2,500 to 3,500 (Ecopiles 4 and 1, respectively) in June 2020 and 1,450 to 2,650 (Ecopiles 5 and 2, respectively) in November 2020. Ecopile 1 showed the highest number of ASVs in the first two temporal samples (3,000 and 3,500), compared to the rest of the Ecopiles. However, in the last sampling time, its number of observed ASVs decreased to 1,900 which contrasts with the tendency of the remaining Ecopiles, whose numbers progressively increased along the timepoints. In addition, the Shannon index of each Ecopile increased over time. To evaluate the differences in diversity during the bioremediation process, a Kruskal-Wallis test of the Shannon indexes was performed. The results are summarized on Figure 1. At the beginning of the bioremediation process (December 2019), most of the Ecopiles (2, 3, 4, 6 and 7) showed a Shannon diversity index of 7.59 (on average), while in the second and third period, this value increases to 9.18 and 9.38, respectively, indicating an increase in diversity. However, Shannon’s values progressively decreased in the Ecopiles 1, and 5; from 9.73 to 7.96 in Ecopile 1 and from 9.37 to 6.96 in Ecopile 5. Samples from June and November tend to show fewer differences (Figure 1). These results show that in most Ecopiles an increment in biodiversity was observed along the bioremediation process.

**Figure 2:**
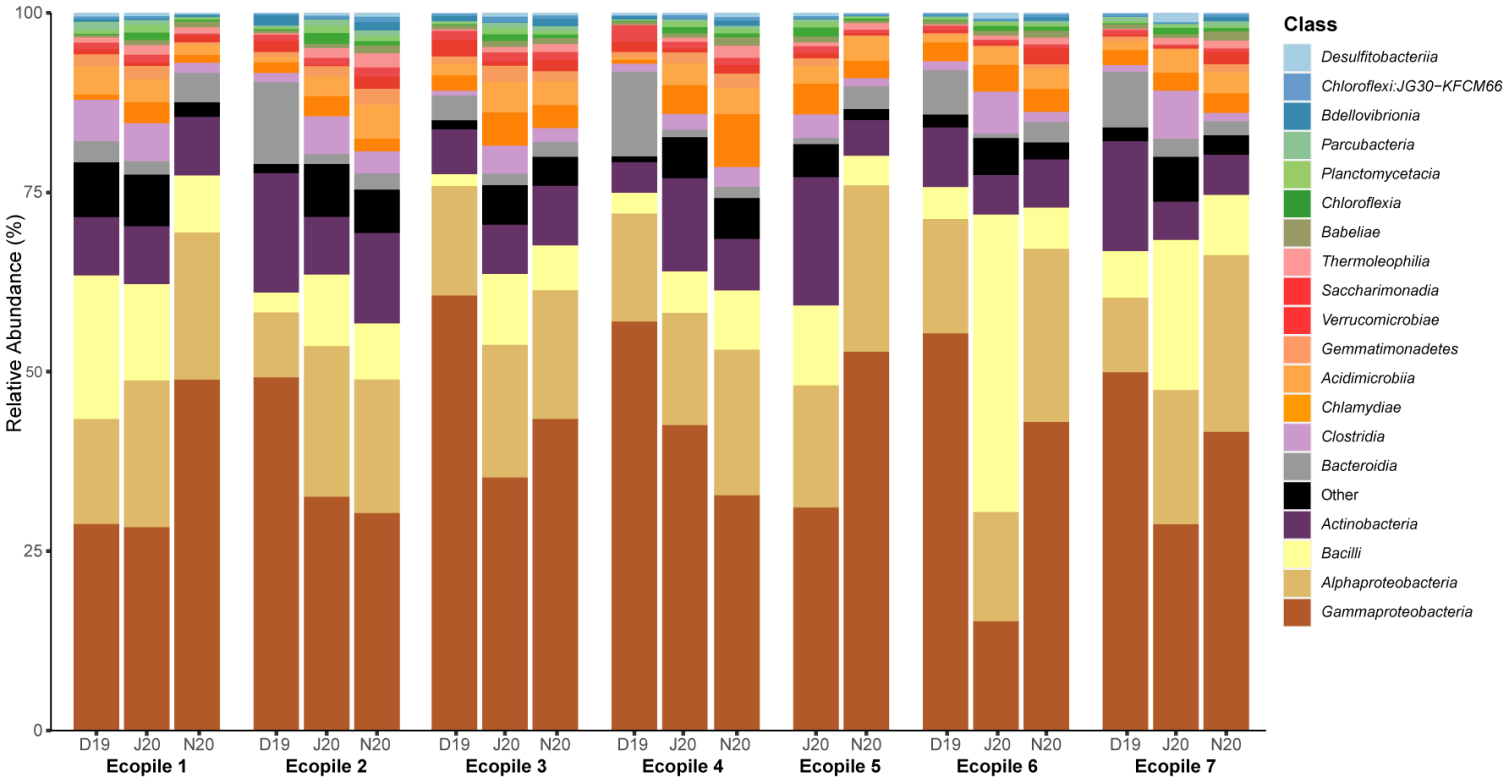
Bacterial relative abundances at the level of class for the different Ecopiles at the three sampling times. Relative bacterial ASVs abundance at the level of class of the Ecopiles at each of the timepoints for the 20 most abundant classes. The remaining classes were grouped under the category Other. The barplots represent the sum of ASVs from the three replicates.

The taxonomic profiles of the seven Ecopiles along the bioremediation process (Figure 2) show two different patterns, as also observed with the Shannon values. In most Ecopiles (2, 3, 4, 6, and 7), a decrease in the percentage of *Proteobacteria* was observed along the bioremediation process. Conversely, in Ecopiles 1 and 5, which showed a declive in the Shannon index at the last timepoint, we also observed an increase in the percentage of *Proteobacteria* and, specifically, in *Gammaproteobacteria*. We also observed that the most abundant classes in the analysis of December 2019 (T2) were *Gammaproteobacteria* (50.16% average relative abundance across Ecopiles), *Alphaproteobacteria* (13.38%), and *Actinobacteria* (9.82%). In June 2020 (T4), *Gammaproteobacteria* (30.56%), *Alphaproteobacteria* (18.07%), and *Bacilli* (16.08%) dominated in the Ecopiles. While in November 2020 (T5), *Gammaproteobacteria* (41.84%), *Alphaproteobacteria* (21.33%), and *Actinobacteria* (7.65%) were the most represented classes. As a general tendency across the Ecopiles, the data show that *Gammaproteobacteria* progressively decreased along the timepoints in Ecopiles 2 and 4, while in Ecopiles 3, 6, and 7 decreased in June and then slightly increased in November, and it increased in Ecopile 1. At the same time, *Alphaproteobacteria* slightly increased or remained similar along the sampling times; *Bacilli* increased in June 2020 and then returned to December 2020’s numbers and *Actinobacteria* did not change along the bioremediation process. Moreover, the classes *Chlamydiae* and *Acidimicrobiia* doubled their numbers comparing December 2019 with November 2020, 1.59 and 1.51% to 3.11, and 3.17%, respectively.

At the genus level (Supplementary Table S2), the most abundant taxa in the analysis of December 2019 were *Pseudomonas* (16.59% average relative abundance across Ecopiles), *Paeniglutamicibacter* (4.27%), and *Rheinheimera* (3.65%). On the other hand, in the analysis of June 2020, *Luteimonas*, *Bacillus,* and *Lysobacter* were the most abundant (8.16, 4.66, and 3.02%, respectively). Finally, in November 2020, *Pseudomonas*, *Silanimonas,* and *Rheinheimera* dominated the Ecopiles (5.69, 5.66, and 3.05%, respectively). The taxonomic profile of the soil used to build the Ecopiles (T0), at the class and genus level, is shown in Supplementary Figure S3.

**Figure 3:**
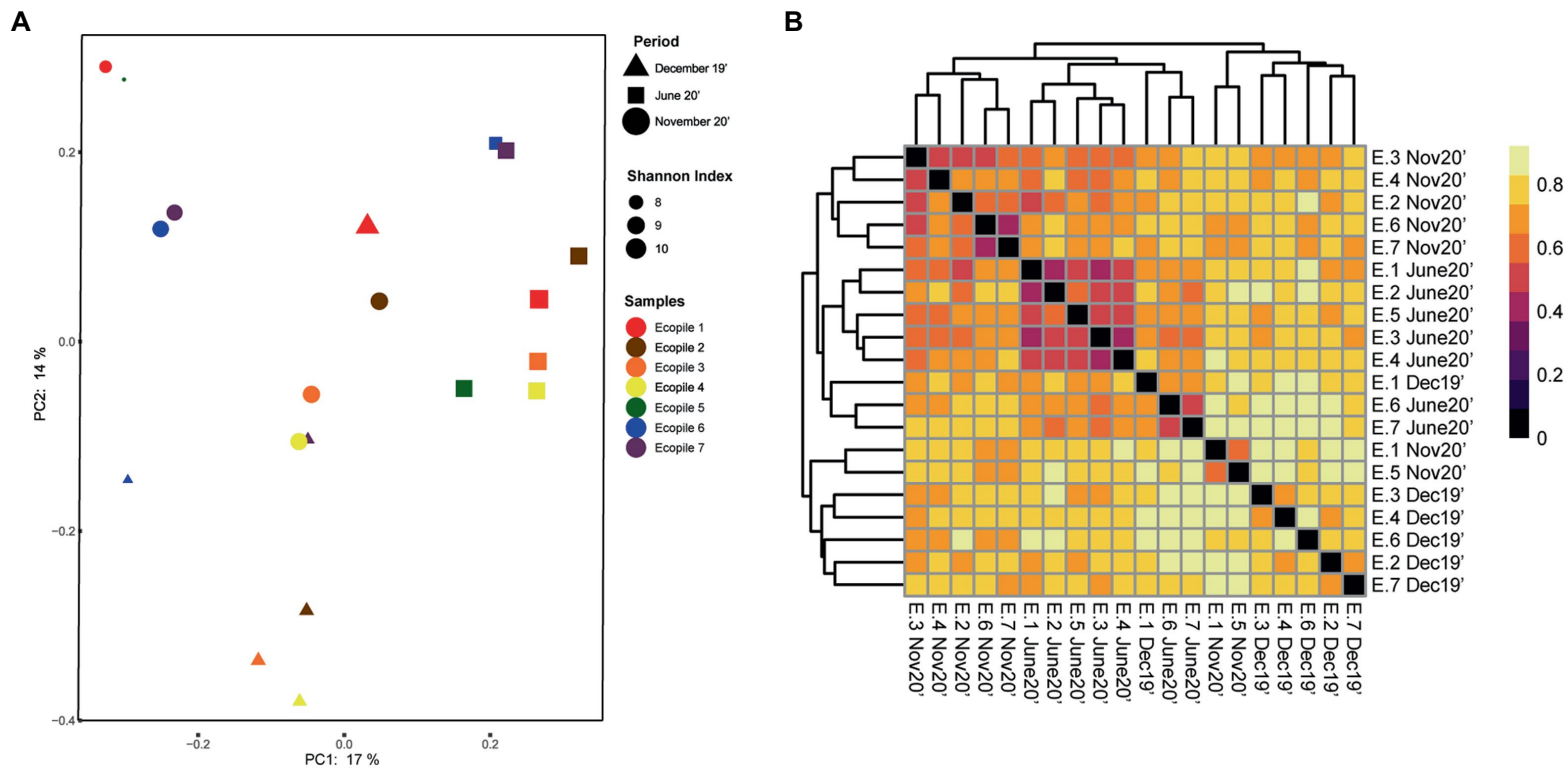
Clustering analysis of the bacterial communities within Ecopiles at the different timepoints. **(A)** Principal coordinate analysis (PCoA) of Ecopiles using Bray-Curtis distances. Colors according to Ecopile, shapes according to timepoint and size according to Shannon index. Averages for each ecopile and sampling time are represented. **(B)** Heatmap of the hierarchical clustering of Ecopiles using Bray-Curtis distances and dendrogram. Color scale indicates Bray-Curtis distances.

To observe differences regarding the beta-diversity between samples, a PCoA ordination plot was performed using Bray-Curtis distances (Figure 3A), where the two first principal components (PCs) explain the 17 and 14% of the observed variance, respectively. The results show that some of December’s samples cluster together (Ecopiles 2, 3, 4, and 6), while all of June’s and part of November’s (Ecopiles 1, 2, 6, and 7) do. This clustering pattern is supported by the hierarchical clustering analysis (Figure 3B), where most samples clustered depending on timepoint sampling with the exceptions of Ecopile 1 on December’s sampling, Ecopiles 6 and 7 in June’s, and Ecopiles 1 and 5 in November’s sampling. There are a few groups that show smaller Bray-Curtis distances, the one formed by June’s samples of Ecopiles 1, 2, 3, 4, and 5 is the largest. Ecopiles 6 and 7 form two groups in the samples of November 2020 and June 2020. Both figures clearly show that samples tend to cluster by sampling time instead of by Ecopile.

**Figure 4:**
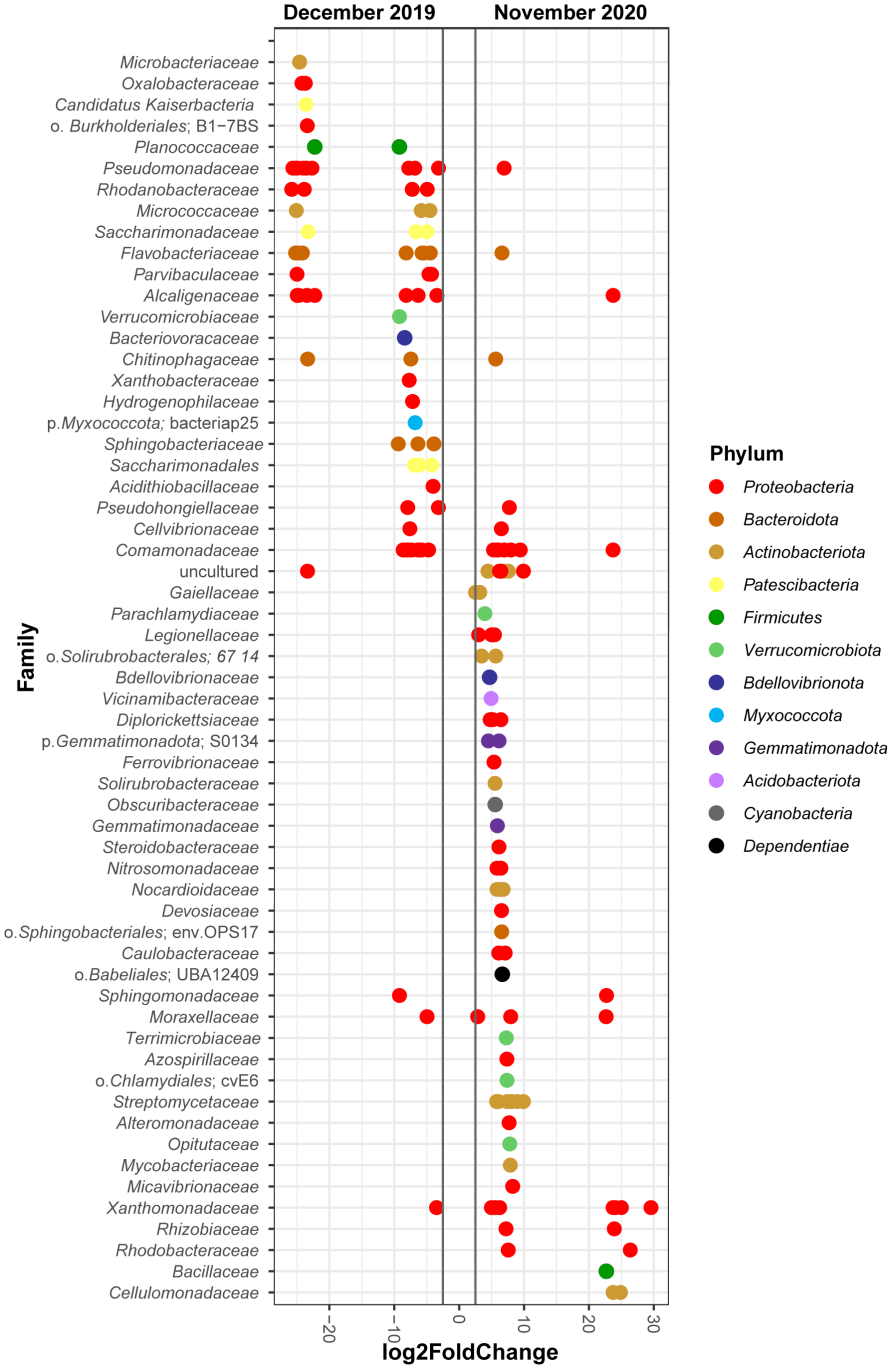
Differential abundance analysis of bacterial ASVs. The analysis shows the log2FoldChange of individual ASVs from the different bacterial families that significantly changed comparing the T2 and T5 timepoints (December 2019 and November 2020, respectively, *p* adjusted value <0.01). Colors according to the phylum to which the represented ASVs and families belong.

In order to determine the populations showing the higher variation over time, we used DESeq2 to compare at the family level the bacterial communities in December 2019 (T2) and November 2020 (T5) (Figure 4). The results show that the phylum with more changes was *Proteobacteria,* with 36 of its ASVs showing a differential increase in December 2019, and 39 in November 2020. The families that had the biggest log2FoldChange in the first timepoint were *Microbacteriaceae*, *Pseudomonadaceae*, *Rhodanobacteraceae*, *Flavobacteriaceae*, or *Alcaligenaceae* among others. In contrast, *Xanthomonadaceae*, *Rhodobacteraceae*, *Bacillaceae*, or *Cellulomonadaceae* were the families that showed the highest differential abundance in the last timepoint. Besides, ASVs from the phyla *Acidobacteriota*, *Cyanobacteria*, *Dependentiae*, *Gemmatimonadota*, and *Verrucomicrobiota* only appeared at significant levels at the last sampling point (November 2020, T5), while *Myxococcota* and *Patescibacteria* show the opposite pattern, as they only were abundant at the first sampling time (December 2019, T2).

**Figure 5:**
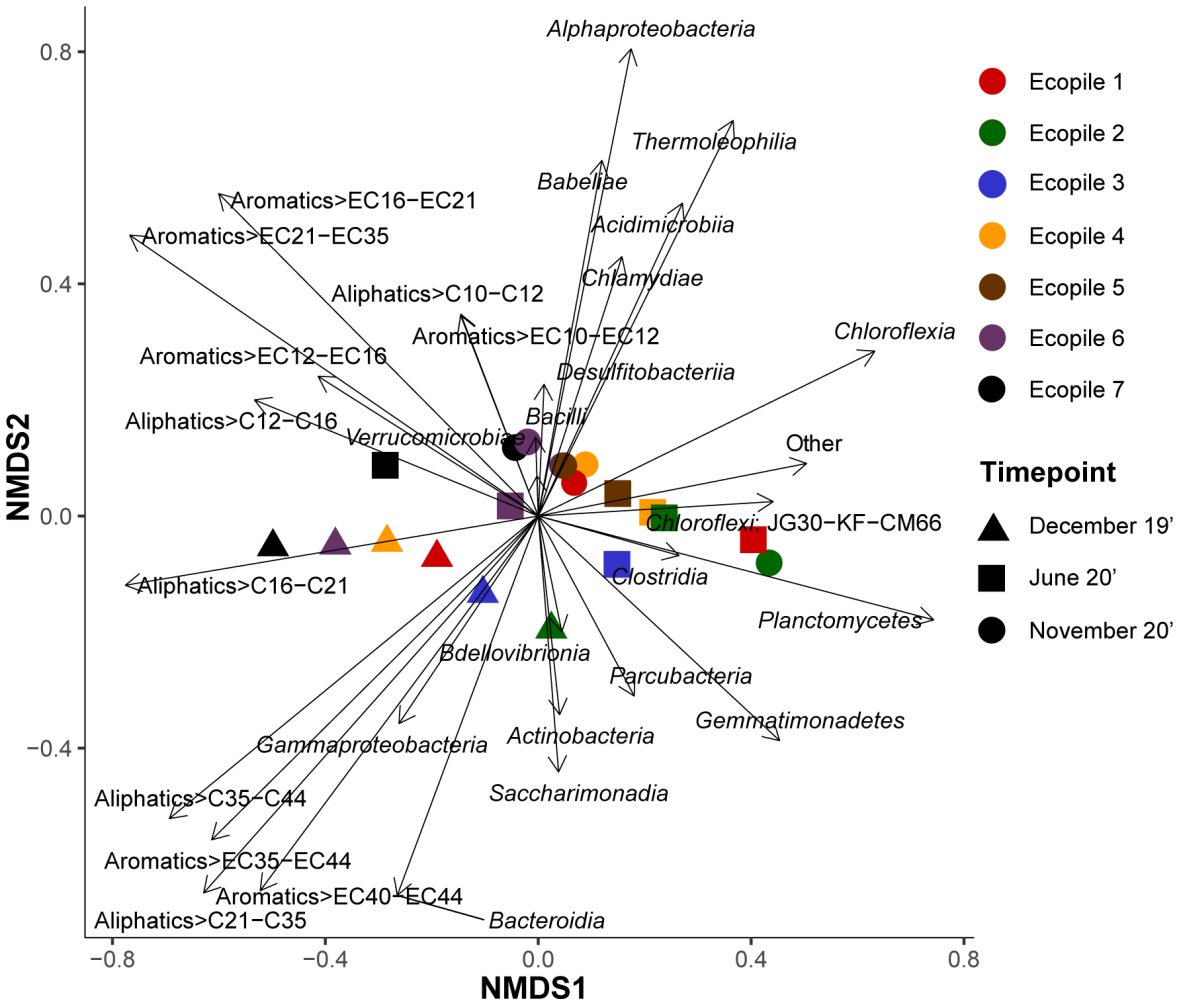
Non-metric multidimensional scaling (NMDS) analysis using Bray-Curtis distance matrix. NMDS plot where the shapes represent the samples according to sampling times and colors according to the Ecopile. Bacterial classes and contaminants were used as explanatory variables driving the Ecopiles distribution pattern.

To further explore the differences between samples based on the bacterial populations and the concentrations of petroleum hydrocarbons, an NMDS analysis was carried out. The results of the analysis (Figure 5) show that *Gammaproteobacteria* slightly justifies the first temporal samplings (Ecopiles 1, 2, 4, 6, and 7) and specially explains December 2019 Ecopile 3 sample. The distribution pattern of these Ecopiles is also explained by the long-chain aliphatic fraction >C_35_-C_44_ and HMW aromatic fractions >EC_35_-EC_44_ and >EC_40_-EC_44_. Similarly, *Bdellovibrionia*, *Actinobacteria,* and *Saccharimonadia* explain December’s Ecopile 2 sample (least polluted sample for aliphatics and second least polluted for aromatics in December 2019, Supplementary Table S1), and middle molecular weight aliphatic fraction >C_16_-C_21_ explain December’s 2019 samples of Ecopiles 4, 6, and 7.

At the same time, some of June’s samples (Ecopiles 1, 2, 3, 4, and 5) and November’s Ecopile 2 sample (the sample from November 2020 contains the lowest quantity of aromatics and the second lowest in aliphatics, Supplementary Table S1) are associated with *Chloroflexia*, *Clostridia,* and *Planctomycetes*, while the middle molecular weight aliphatics (>C_16_-C_21_) are negatively associated with these samples. Conversely, *Verrucomicrobia* and several low, middle, and high molecular weight pollutants (>C_12_-C_16_, >EC_12_-EC_16_, and >EC_21_-EC_35_) explain June’s samples of Ecopiles 6 and 7 (most polluted samples for both hydrocarbon types in June 2020, Supplementary Table S1). Furthermore, most of November’s samples (Ecopiles 1, 3, 4, 5, 6, and 7) are associated with the classes *Acidimicrobiia*, *Alphaproteobacteria*, *Babeliae*, *Bacilli*, *Desulfitobacteriia,* and *Thermoleophilia*. Besides, the samples of Ecopiles 6 and 7 at the last timepoint are explained by two groups of LMW pollutants (>C_10_-_12_ and > EC_10_-_12_). Moreover, the NMDS analysis showed a stress value of 0.0174 and correlation statistics of 0.99 and 1 for linear fit and non-linear fit, respectively.

### 3.3. Evolution of the fungal community along the bioremediation process

**Figure 6:**
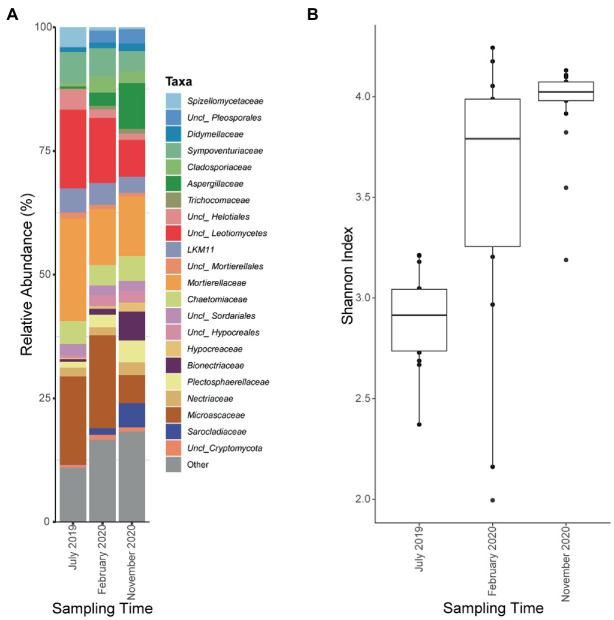
Fungal relative abundances at the level of class and family and Shannon indexes of Ecopiles at the three sampling times. **(A)** Relative ASVs abundance at the level of class of the Ecopiles at each of the timepoints. Low abundance taxa were grouped under the category Other. The barplots represent the average of ASVs from Ecopiles. **(B)** Evolution of Shannon alpha-diversity indexes within Ecopiles along sampling time.

18S rRNA amplicon was also sequenced in the seven Ecopiles throughout the experimental period. An average of 142,000 reads per sample were sequenced of which about 81% were conserved after DADA2 analysis grouped into 8,169 ASVs. After eliminating those ASVs of low abundance and those that had not been taxonomically assigned to the kingdom Fungi, about 68,000 reads on average per sample were conserved, and clustered into 456 ASVs (Supplementary Figure S4). As observed in the analysis performed for bacteria, an increase in the number of ASVs observed over time was detected. Specifically, the minimum value was found in the July 2019 (T1) sample in Ecopile 7 with an average of 85 ASVs and the maximum value was found in the November 2020 (T5) samples with several ASVs greater than 250. This increase in the diversity of each ecopile was supported by Shannon indices using sampling time as a variable (Figure 6B). Thus, the mean index in July 2019 (T1) is 2.91, which increases in February 2020 (T3) to 3.79 and reaching its maximum value at the end of the bioremediation process, in November 2020 (T5) with an index of 4.02. These values show significant differences using the Wilcoxon signed-rank test with a value of p adjustment method of Benjamini-Hochberg (adjusted *p* value <0.01).

**Figure 7:**
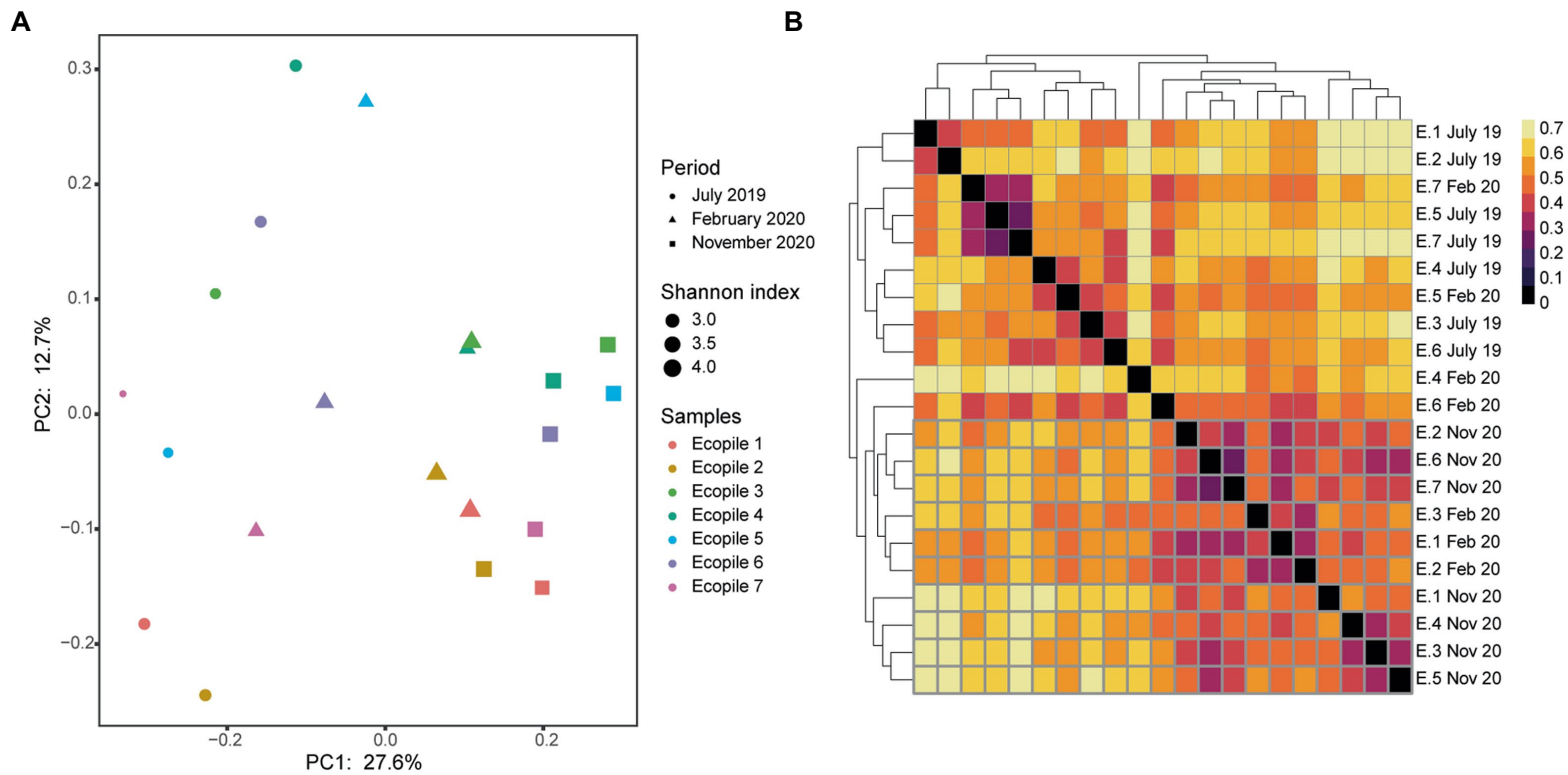
Clustering analysis of the fungal communities within Ecopiles along the different timepoints. **(A)** Principal coordinate analysis (PCoA) of Ecopiles using Bray-Curtis distances. Colors according to Ecopile, shapes according to timepoint and size according to Shannon index. Averages are represented for each ecopile and sampling time. **(B)** Heatmap of the hierarchical clustering of Ecopiles using Bray-Curtis distances and dendrogram. Color scale indicates Bray-Curtis distances.

Regarding the variation in the taxonomic profile, Figure 6A shows how the most abundant families in 2019 such as *Microascaceae* (17%) and *Mortierellaceae* (20.6%), or individuals of the class *Leotiomycetes* (15%) and *Helotiales* (4%) see their presence decrease over time in favor of individuals of the family *Aspergillaceae* (9%), *Bionectriaceae* (5%), and *Sarocladiaceae* (4.8%) among others. This population shift is supported by beta-diversity analysis using Bray-Curtis distances. The PCoA analysis (Figure 7A) shows a clear separation of the Ecopiles over time along the PC1 axis (explaining 27.6% of the variance). Such behavior is also seen in the hierarchical clustering of distances (Figure 7B) and in the PERMANOVA analysis of distances grouping samples by sampling (adjusted *p* value <0.01).

**Figure 8:**
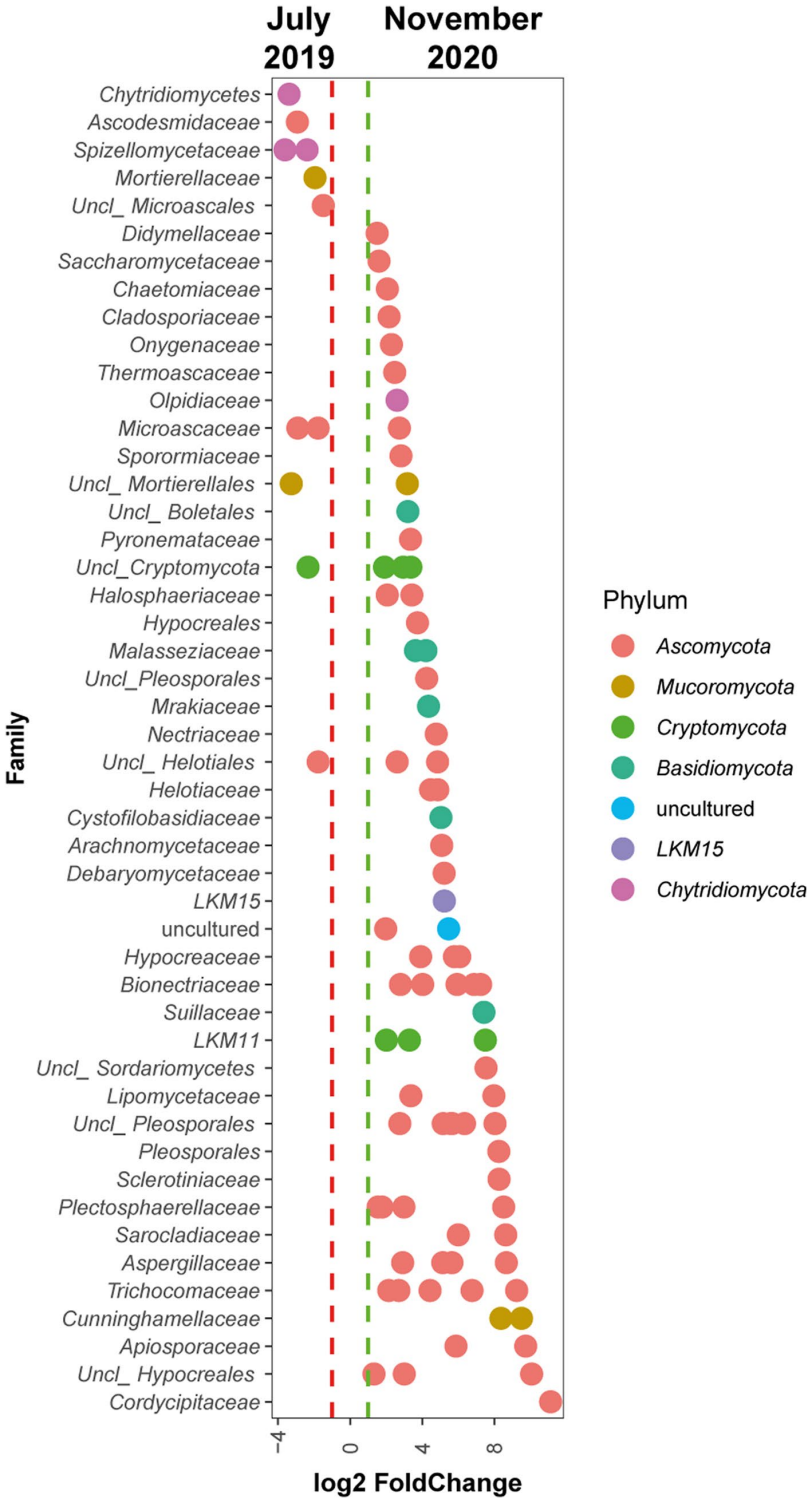
Differential abundance analysis of fungal ASVs. The analysis shows the log2Fold Change of individual ASVs from the different fungal families that significantly changed comparing July 2019 and November 2020 sampling times (*p* adjusted value <0.01).

Comparison using DESeq2 of community status in July 2019 (T1) with respect to November 2020(T5) (Figure 8) showed that the Phyla showing the greatest differences were those corresponding to *Chytridiomycota*, enriched at T1 and versus members of *Ascomycota* and *Basidiomycota*, mainly enriched at T5. ASVs of the class *Chytridiomycetes* and the family *Spizellomycetaceae* were displaced over time compared to individuals of the families *Cordycipitaceae*, *Apiosporaceae*, *Trichocomaceae*, *Aspergillaceae*, *Sarocladiaceae*, and *Sclerotiniaceae* of the phylum *Ascomycota* and individuals of the families *Malasseziaceae*, *Cystofilobasidiaceae,* and *Mrakiaceae* of the phylum Basidiomycota. It is also noteworthy that within the phylum *Mucoromycota*, populations of individuals of the family *Mortierellaceae* decrease over time compared to members of the family *Cunninghamellaceae*.

## 4. Discussion

Ecopiling represents a cost-effective technique that combines multiple bioremediation approaches to amend hydrocarbon polluted soils (Germaine et al., 2015). Our study clearly shows the progression of the bioremediation process in Ecopiles. The initial TPHs of the polluted soils were degraded, reaching 99% for aliphatics and 88% for aromatics at the end of the process (Table 1). As previous studies show, the slower and lower degradation of aromatics could be caused by the fact that they include complex PAHs, some of which are considered persistent organic pollutants with low bioavailability (Abdel-Shafy and Mansour, 2016; Crampon et al., 2018) and are therefore highly recalcitrant. Conversely, aliphatic and LMW aromatic compounds usually require less time to be degraded and are preferred by bacteria (Sutton et al., 2013; Shahi et al., 2017). Moreover, the most significant part of the degradation process took place during the first months, between the start of the ecopiling process in May 2019 and June 2020 (Table 1). Higher degradation rates were observed in the outer layers of the Ecopile with lower degradation rates with increasing soil depth. This is likely due to the solid rhizoremediation action in the outer layers due to the abundance of plant roots. The consequence of this it is the maximum height of Ecopiles should be limited to 1.5 m, the typical depth till which rye-grass roots grow.

Regarding the bacterial communities of the Ecopiles, the number of detected ASVs (Figure 2) in June 2020 (3,000 on average) nearly doubled the values of December 2019 (1,890 on average). These values are in line with previous studies that highlighted how fewer ASVs are detected in environments polluted with petroleum hydrocarbons (Gałązka et al., 2018; Ruley et al., 2020). The alpha-diversity measured by the Shannon index (Figures 1, 3) was significantly higher in June and November 2020 timepoints (9.44, 8.98 of average across Ecopiles, respectively), whose samples had lower TPHs’ levels than in December 2019 (7.99). These results also agree with previous studies, which show that contaminated soils have a lower bacterial diversity (Peng et al., 2015; Gałązka et al., 2018; Yan et al., 2018; Ruley et al., 2020). Moreover, the higher values in terms of the Shannon index observed in June 2020 could be explained by the fact that microbial biomass and activity are higher during summer (Jangid et al., 2008). Taken together, these results indicate that bacterial diversity has increased in Ecopiles along the biodegradation process, suggesting that ecopiling not only eliminate pollutants such as TPHs, but also contributes to soil restoration by increasing bacterial biodiversity.

Regarding the observed relative abundances (Figure 2), the distribution in December 2019 timepoint for most of the Ecopiles (2, 3, 4, 6, and 7) was similar to the distribution of the starting of the process, May 2019 (Wang et al., 2021), with *Gammaproteobacteria* representing almost 50% of the abundance, *Alphaproteobacteria* with a 20%, and *Actinobacteria, Bacteroidia* and *Bacilli* with values near 10%. Furthermore, specific patterns were found in previous longitudinal bioremediation studies, such as the predominance and changes in the relative abundance of *Gammaproteobacteria*. It has been suggested that it could be caused by the variations in TPHs’ levels, as some strains of this class have biodegradative potential of aliphatic and aromatic compounds, which would promote their growth in numbers (Sutton et al., 2013; Tardif et al., 2016). The stability shown by the class *Alphaproteobacteria* along the bioremediation process, or the reduced presence of *Firmicutes* (classes *Bacilli* and *Clostridia*) when there were higher concentrations of pollutants, and their subsequent increase might be explained by the fact that they are commonly found in non-polluted soils (Militon et al., 2010). A similar pattern is observed regarding *Acidobacteria* and *Verrucomicrobia,* which increased their abundance at the end of the process (Janssen Peter, 2006; Jangid et al., 2008). At the genus level (Supplementary Table S10), a few bacterial genera represented a significant part of the total relative abundance in the first samplings (16.59% of *Pseudomonas*, 4.27% of *Paeniglutamicibacter,* and 3.56% of *Rheinheimera*). While at the last sampling time, those percentages decreased, and most genera were present at low relative abundances (all of them under 6%), the pattern typically followed by soil bacterial communities (Bickel and Or, 2021). Also, the percentage of genera grouped under “Other” rose from 51.243% in December 2019 to 61.79% in November 2020 (Supplementary Table S2), highlighting again how taxa show lower relative abundances in soils with fewer hydrocarbon pollutants. Moreover, some of these patterns were also observed in the differential abundance analysis, which shows how *Proteobacteria* is the phylum whose families changed the most, which could be caused by the TPHs’ variations (Vivas et al., 2008; Sutton et al., 2013; Gałązka et al., 2018). The analysis also highlights how families with known hydrocarbon degrading potential, like *Alcaligenaceae* or *Pseudomonadaceae* (Ruiz et al., 2021), displayed remarkable log2FoldChange values in December 2019, when TPHs’ numbers were high. Conversely, ASVs from families of the phyla *Acidobacteriota*, and some *Actinobacteriota* or *Firmicutes,* appeared at the end of the process, when there were substantially less pollutants than at the beginning of the bioremediation process. The mentioned phyla are typical of oligotrophic soils and contain genus like *Bacillus* or *Streptomyces* (Janssen Peter, 2006; Delgado-Baquerizo et al., 2018). All these results point to a bacterial succession in which bacteria typical of clean oligotrophic soils substitute bacteria with a potential for TPHs biodegradation. These results highlight the soil restoration process that occurs at Ecopiles.

Following the bacterial diversity profiles of the samples, Ecopiles 1 and 5 showed a similar pattern, in which the Shannon index decreased progressively (Figure 3), and the relative abundance of bacteria are almost identical at the class level (Figure 2). Similarly, the PCoA and the hierarchical clustering analysis (Figures 5A,B) grouped June’s and November’s samples of these Ecopiles. Ecopiles 6 and 7 also showed similar profiles and progression in the mentioned analyses. This can be explained by the fact that both were the most polluted Ecopiles for aliphatics and aromatics in all the timepoints (except for aliphatics in Ecopile 6 at the final timepoint) (Table 1).

Regarding the results of the NMDS analysis (Figure 6), it is interesting to note that the first temporal samples (December 2019) are grouped in the lower left quadrant, associated with vectors representing the higher molecular weight pollutants: aliphatics (C_16_ to C_35_) and aromatics (C_35_ to C_44_). Most of these samples were also associated with *Gammaproteobacteria*, a group that has been previously associated with the degradation of hydrocarbon pollutants (Militon et al., 2010; Sutton et al., 2013). Interestingly, the December 2019 Ecopile 2 sample was associated with *Actinobacteria*, and *Saccharimonadia*, and was the least polluted sample of the initial timepoint for both aliphatics and aromatics (Table 1), which could promote the abundance of some of these classes, as previously reported (Borowik and Wyszkowska, 2018). All other pollutants presented vectors pointing to the upper left quadrant, resulting that all the vectors for TPHs are in the left half of the graph. Only samples from Ecopiles 6 and 7 in June 2020, the most polluted group in this quadrant and seem to be associated with low molecular weight pollutants and with *Verrucomicrobia*. All the other samples from June 2020 and all samples from November 2020, group in the right half of the graph. These samples were associated with *Chloroflexia*, *Clostridia,* and *Planctomycetes*, while most of November’s samples (Ecopiles 1, 3, 4, 5, 6, and 7) were explained by the classes *Acidimicrobiia*, *Alphaproteobacteria*, *Babeliae*, *Bacilli*, *Desulfitobacteriia,* and *Thermoleophilia,* which are groups typical of oligotrophic soils (Borowik and Wyszkowska, 2018; Delgado-Baquerizo et al., 2018).

A succession was also observed for the fungal community. This succession is very similar to the bacterial succession showed above since it is also accompanied by an increase in alfa diversity, as clearly shown by the increase in the Shannon index. The changes in fungal populations are also supported by beta-diversity analysis, which shows a clear separation between T1 samples and most samples from T3 and T5. These differences are highlighted by clustering analysis, where samples from year 2020, clustered separately from 2019 samples. Finally, microdiversity analysis at the level of individual ASVs, show that fungal populations abundant at the beginning of the ecopiling process are substituted for other fungal populations at the end of the process. Although it is difficult to co-relate specific ASVs with soil quality, taken together, the presented results suggest a microbial succession leading to microbial communities typical of clean soils.

It can be concluded that the Ecopiling method was successful in the biodegradation of hydrocarbon pollutants. TPHs levels decreased by more than 90% on average during the 18 months of treatment. Furthermore, most of TPHs removal was achieved during the first months of treatment. This decrease in soil pollution had a profound effect on the soil’s microbial communities. Along the bioremediation process, an increase in microbial biodiversity was observed. This increase in biodiversity was evident both for bacterial and fungal communities. The observed results indicate a microbial succession, from microbial communities typical of polluted soils to communities which indicate clean soils. Therefore, ecopiling contributes to both bioremediation of hydrocarbon contaminated soils and restoration of soil microbiota and hence soil quality.

## Data availability statement

The datasets presented in this study can be found in online repositories. The names of the repository/repositories and accession number(s) can be found at: https://www.ncbi.nlm.nih.gov/, PRJNA925022.

## Author contributions

RM-C, RC, MW, EB-R, DDu, and MR-N carried out the microbiome and metagenome analysis. RC and MW prepared samples for chemical analysis. KG set up the Ecopiling experiment. MM, DDo, DG-S, MR-N, KG, and RR were involved in the experimental design and preparation of the manuscript. All authors contributed to the article and approved the submitted version.

## Funding

This project has been funded by the European Union’s Horizon 2020 research and innovation programme “GREENER” under grant agreement number 826312. MW has been funded by the Irish Research Council under the Government of Ireland Postgraduate Scholarship Programme project number GOIPG/2021/1238.

## Conflict of interest

The authors declare that the research was conducted in the absence of any commercial or financial relationships that could be construed as a potential conflict of interest.

## Publisher’s note

All claims expressed in this article are solely those of the authors and do not necessarily represent those of their affiliated organizations, or those of the publisher, the editors and the reviewers. Any product that may be evaluated in this article, or claim that may be made by its manufacturer, is not guaranteed or endorsed by the publisher.
