# Supplementary material for: Field scale biodegradation of total petroleum hydrocarbons and soil restoration by Ecopiles: microbiological analysis of the process
Source: Front Microbiol. 2023 Apr 21;14:1158130. doi: 10.3389/fmicb.2023.1158130 (PMC10160625; doi:10.3389/fmicb.2023.1158130)
Supplement: Supplementary file 1 [file Data_Sheet_1.pdf]

Supplementary Material

Field scale biodegradation of Total Petroleum Hydrocarbons and soil restoration by Ecopiles. Microbiological analysis of the process.

Ruben Martínez-Cuesta[1], Robert Conlon[2], Mutian Wang[2], Esther Blanco-Romero[1], David Durán[1], Miguel Redondo-Nieto[1], David Dowling[2], Daniel Garrido-Sanz[1], Marta Martin[1], Kieran Germaine[2], and Rafael Rivilla[1*]

[1]Departamento de Biología, Universidad Autónoma de Madrid, Madrid, Spain.

[2]EnviroCore, Dargan Research Centre, South East Technological University, Carlow, Ireland.

*** Correspondence:** Corresponding Author: rafael.rivilla@uam.es

Table S1. Chemical analysis of the petroleum hydrocarbons measured in the studied Ecopiles and sampling times along the bioremediation process.

Samples	Petroleum hydrocarbon fractions (µg/kg)											
	Aliphatics $>C_{10}$-C_{12}	Aliphatics $>C_{12}$-C_{16}	Aliphatics $>C_{16}$-C_{21}	Aliphatics $>C_{21}$-C_{35}	Aliphatics $>C_{35}$-C_{44}	Total Aliphatics$>C_{12}$-C_{44}	Aromatics $>EC_{10}$-EC_{12}	Aromatics $>EC_{12}$-EC_{16}	Aromatics $>EC_{16}$-EC_{21}	Aromatics $>EC_{21}$-EC_{35}	Aromatics $>EC_{35}$-EC_{44}	Total Aromatics$>EC_{12}$-EC_{44}
E.1 Dec19	0	3,960	35,600	414,500	158,500	612,500	0	2,575	34,450	552,500	317,000	906,000
E.2 Dec19	0	4,210	22,950	188,000	79,400	295,000	0	2,525	19,100	213,000	124,500	359,000
E.3 Dec19	0	4,530	30,250	317,500	127,500	480,000	0	2,360	26,250	387,500	227,000	643,500
E.4 Dec19	0	9,280	55,750	573,500	227,500	866,000	0	4,450	51,100	775,000	448,000	1,280,000
E.5 Dec19	0	8,545	56,700	618,500	245,000	928,500	0	2,795	41,100	690,500	339,500	118,000
E.6 Dec19	0	14,750	86,400	930,500	386,500	1,415,000	0	9,045	81,900	1,017,000	562,000	1,670,000
E.7 Dec19	67	31,450	137,500	1,265,000	535,500	1,970,000	45	20,550	145,500	1,545,000	935,000	2,640,000
E.1 June20	0	0	4,050	4,430	0	11,200	0	1,415	15,650	143,000	20,150	176,000
E.2 June20	0	3,010	9,335	7,730	1,260	7,910	0	3,260	13,550	115,500	16,600	185,000
E.3 June20	0	5,620	35,050	80,175	22,800	20,300	0	5,255	28,450	276,500	42,000	152,000
E.4 June20	0	2,915	14,000	14,835	1,850	22,800	0	4,785	31,550	258,000	45,400	145,000
E.5 June20	0	2,540	11,350	9,780	1,815	22,700	0	4,745	39,700	340,500	65,000	322,000
E.6 June20	4,270	23,500	66,350	198,500	6,415	118,000	1,840	25,900	85,150	527,500	99,050	382,000
E.7 June20	13,200	68,550	150,500	448,300	386,500	27,000	4,655	52,300	131,500	764,000	29,100	326,000
E.1 Nov20	0	0	30,900	32,450	0	66,550	0	0	28,300	454,500	91,650	573,500
E.2 Nov20	0	0	12,930	9,475	0	26,600	0	0	10,760	258,500	46,650	315,500
E.3 Nov20	0	0	17,750	15,900	0	35,200	0	0	35,600	528,000	93,800	658,500
E.4 Nov20	0	0	0	6,955	0	25,000	0	0	36,050	463,000	67,050	566,000
E.5 Nov20	0	3,350	12,265	12,350	0	34,100	0	0	44,400	507,500	96,500	649,000
E.6 Nov20	0	0	8,265	12,100	0	27,550	0	0	63,700	668,500	120,500	854,500
E.7 Nov20	0	0	28,300	21,700	0	53,500	0	0	54,150	724,000	143,000	924,500

Table S2. Relative abundances of bacterial genera along the different timepoints with the average at the end of each one.

Genus	• Samples																						
	**E.1 D19**	**E.2 D19**	**E.3 D19**	**E.4 D19**	**E.6 D19**	**E.7 D19**	**Avg. D19**	**E.1 J20**	**E.2 J20**	**E.3 J20**	**E.4 J20**	**E.5 J20**	**E.6 J20**	**E.7 J20**	**Avg. J20**	**E.1 N20**	**E.2 N20**	**E.3 N20**	**E.4 N20**	**E.5 N20**	**E.6 N20**	**E.7 N20**	**Avg. N20**
Pseudomonas	1.62	18.55	29.3	19.03	18.77	12.26	16.59	0.96	0.35	0.8	1.41	2.45	0.42	1.75	1.16	3.77	1.13	10.12	9.02	1.62	8.09	6.07	5.69
Luteimonas	1.01	2.33	4.24	4.58	0.05	6.09	3.05	8.71	13.08	10.51	14.32	6.56	1.57	2.37	8.16	0.72	2.72	4.06	0.82	0.22	1.57	2.58	1.81
Bacillus	7.67	0.66	0.55	0.65	0.87	1.00	1.90	4.91	3.58	2.53	2.24	2.34	8.85	8.15	4.66	2.75	2.71	2.49	2.83	1.71	2.61	4.94	2.86
f.*Acidithiobacillaceae;* KCM-B-112	8.81	1.08	4.41	1.78	1.42	2.86	3.39	2.08	1.60	2.69	2.09	2.73	2.40	1.52	2.16	0.57	2.54	3.16	1.03	0.44	2.08	2.01	1.69
Rheinheimera	0.46	0.55	0.06	0.02	20.58	0.22	3.65	0.00	0.00	0.00	0.00	0.00	0.00	0.00	0.00	8.75	0.07	0.57	0.05	0.82	4.39	6.69	3.05
Silanimonas	0.10	0.00	0.00	0.00	0.02	0.01	0.02	0.00	0.00	0.00	0.00	0.00	0.00	0.01	0.00	9.68	0.00	0.00	0.00	29.41	0.34	0.23	5.66
Brevundimonas	1.07	1.01	2.36	1.71	8.06	1.80	2.67	0.77	0.82	0.68	0.61	2.21	0.34	0.61	0.86	3.28	0.92	1.30	1.38	2.38	3.48	4.88	2.52
f.*Microbacteriaceae;*	2.02	1.77	2.81	1.76	2.24	0.60	1.87	1.68	1.79	1.31	1.99	1.94	0.91	1.44	1.58	2.55	1.76	1.60	0.85	1.59	1.92	1.26	1.65
Pseudoxanthomonas	0.61	4.05	4.08	2.05	0.66	3.64	2.51	1.54	0.24	1.53	0.70	1.11	0.19	0.12	0.78	0.50	1.95	1.44	3.41	0.56	1.96	2.49	1.76
Lysobacter	0.65	0.73	0.48	0.07	0.09	0.38	0.40	1.85	4.09	2.45	1.29	1.97	1.18	8.33	3.02	0.61	1.23	1.29	1.19	0.05	1.39	0.44	0.88
Paeniglutamicibacter	0.20	8.12	0.15	0.22	2.83	14.13	4.27	0.37	0.13	0.03	0.26	1.30	0.05	0.01	0.31	0.66	0.18	0.40	0.08	0.12	0.14	0.12	0.24
Hydrogenophaga	1.33	0.06	0.06	0.17	2.15	0.04	0.64	0.36	0.41	0.32	0.31	0.40	1.33	1.95	0.72	7.02	0.16	0.49	0.27	9.80	1.27	1.06	2.87
Neochlamydia	0.18	0.79	1.12	0.34	1.16	0.90	0.75	1.32	1.13	2.12	1.78	2.13	2.09	1.50	1.73	0.47	0.82	1.51	4.03	1.41	1.57	1.23	1.58
Rhodanobacter	0.17	2.65	1.67	11.44	0.00	1.39	2.89	0.98	1.09	1.25	2.94	0.10	0.53	0.63	1.08	0.02	0.75	0.80	0.29	0.01	0.14	0.09	0.30
Sphingomonas	0.75	0.61	1.14	0.32	0.75	0.42	0.67	4.10	1.37	2.25	1.74	2.73	0.34	0.27	1.83	0.75	2.48	1.57	1.12	0.88	1.31	1.29	1.34

Nocardioides	0.22	0.21	0.27	0.68	0.03	0.27	0.28	2.08	2.33	2.22	5.51	4.40	0.48	0.66	2.53	0.73	1.50	1.18	0.71	0.62	0.89	0.92	0.94
Parvibaculum	0.88	1.27	3.59	4.73	0.09	0.51	1.85	1.22	3.06	1.82	1.88	0.77	0.65	1.02	1.49	0.06	1.04	1.17	0.25	0.07	0.90	0.82	0.62
Paenibacillus	2.59	0.44	0.17	0.61	0.29	0.63	0.79	2.54	1.76	1.70	0.70	0.83	3.17	2.28	1.85	1.86	1.23	0.77	1.44	0.68	0.61	1.13	1.10
Legionella	0.23	0.56	0.43	1.46	0.34	0.54	0.59	1.03	0.98	1.30	1.40	1.17	2.00	2.38	1.47	0.84	2.07	1.81	1.73	0.88	1.98	2.25	1.65
Other	69.43	54.57	43.13	48.38	39.62	52.32	51.24	63.50	62.20	64.47	58.86	64.88	73.50	65.01	64.63	54.41	74.74	64.26	69.53	46.75	63.37	59.49	61.79

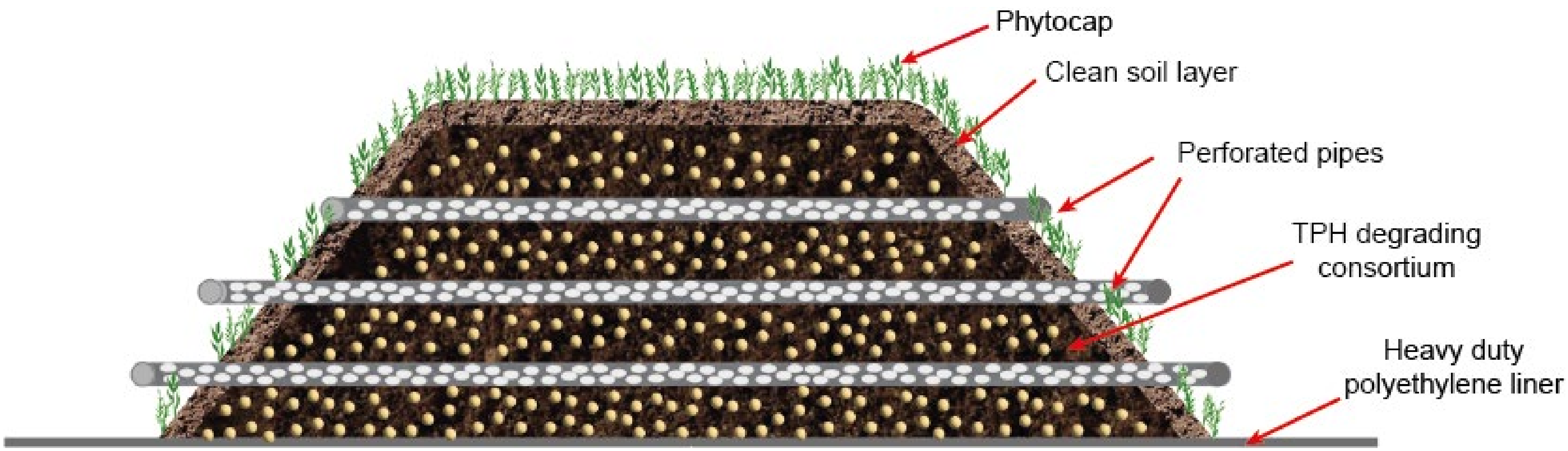

Figure S1. Schematic representation of the ecopile structure. The contaminated soil was stacked in a trapezoidal structure on top of a layer of heavy-duty polyethilene liner up to a height of 2m. Every 0.5 m, 50 mm perforated pipes were inserted to allow passive ventilation of the system. Nutrients were added in the form of nitrogen:phosphorus fertilizer (25:4) and the hydrocarbon degrading consortium was added. Finally, the structure was covered with an uncontaminated layer of soil (~5 cm) on which a mixture of clover and ryegrass seeds were planted.

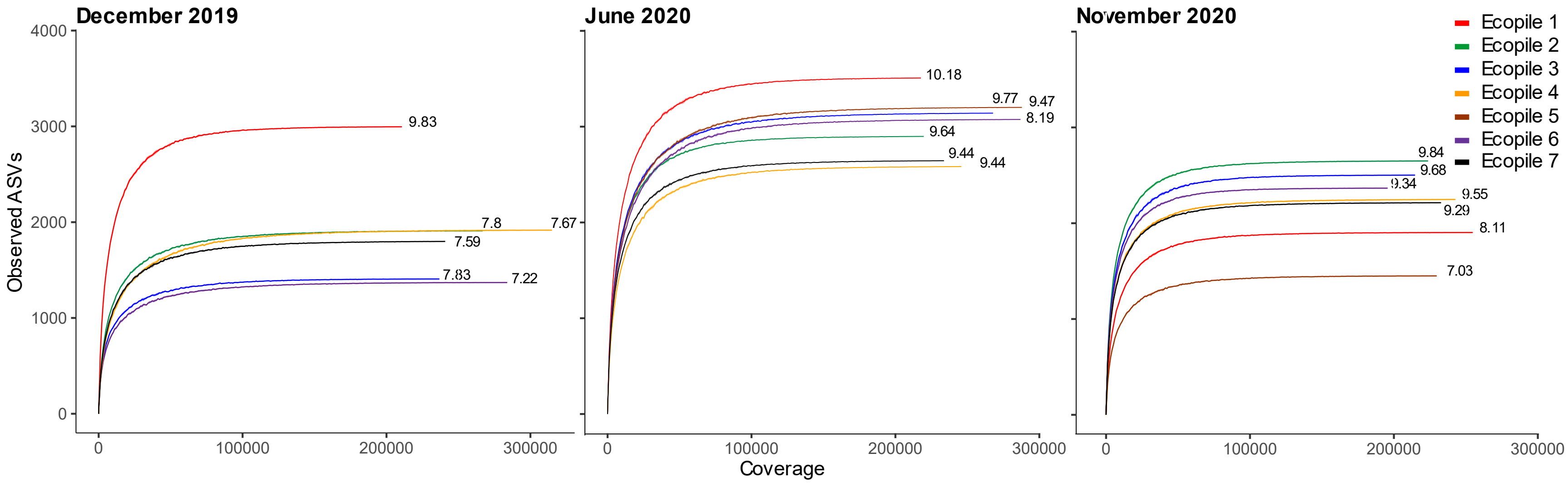

Figure S2. Bacterial rarefaction curves and Shannon index across Ecopiles and timepoints. Rarefaction curves of 16S ASVs observed over the total reads from the different Ecopiles over the three sampling times, December 2019, June 2020 and November 2020. Each curve represents the merged sequences from the three Ecopile replicates. Shannon index values are indicated at the end of each curve, respectively.

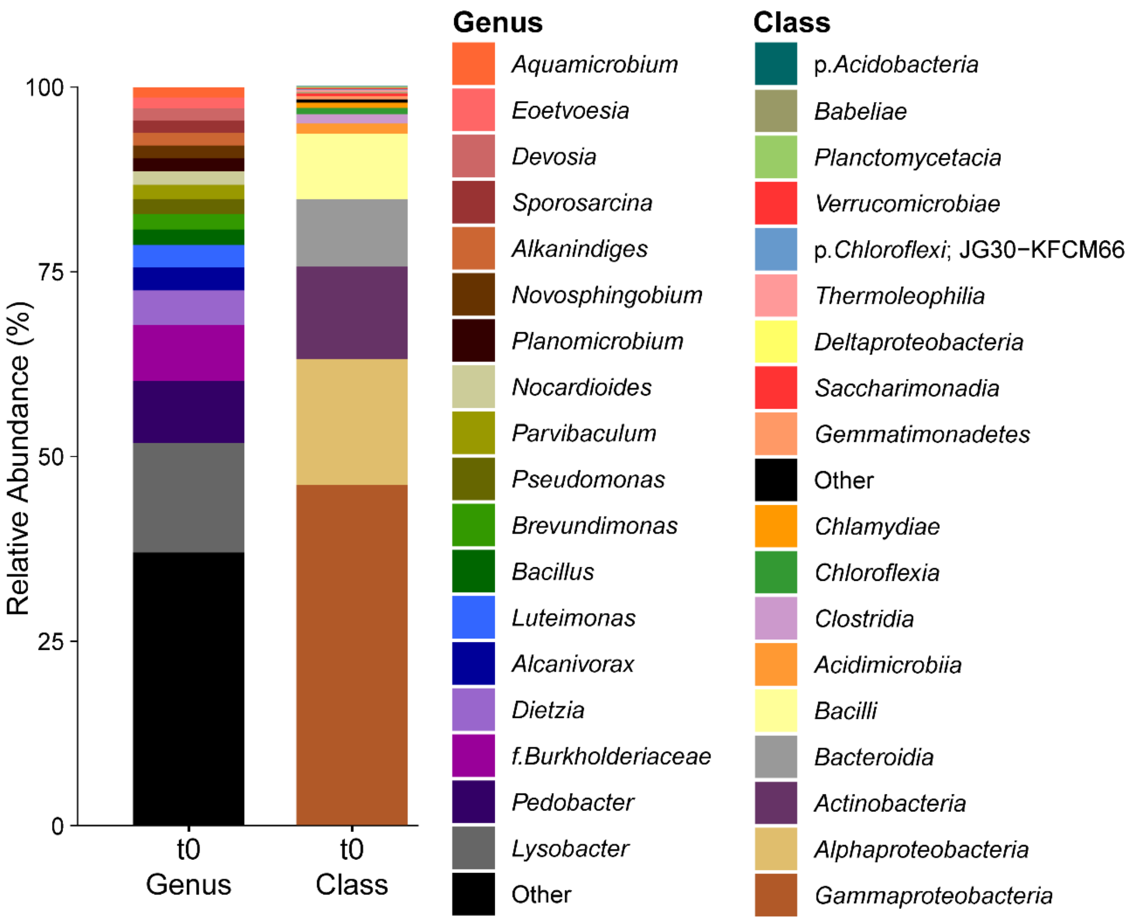

Figure S3. Relative abundances at the levels of genus and class for the t0 sampling time. Barplot represents the average relative abundance of ASVs in the bulk polluted soil used for building the Ecopiles

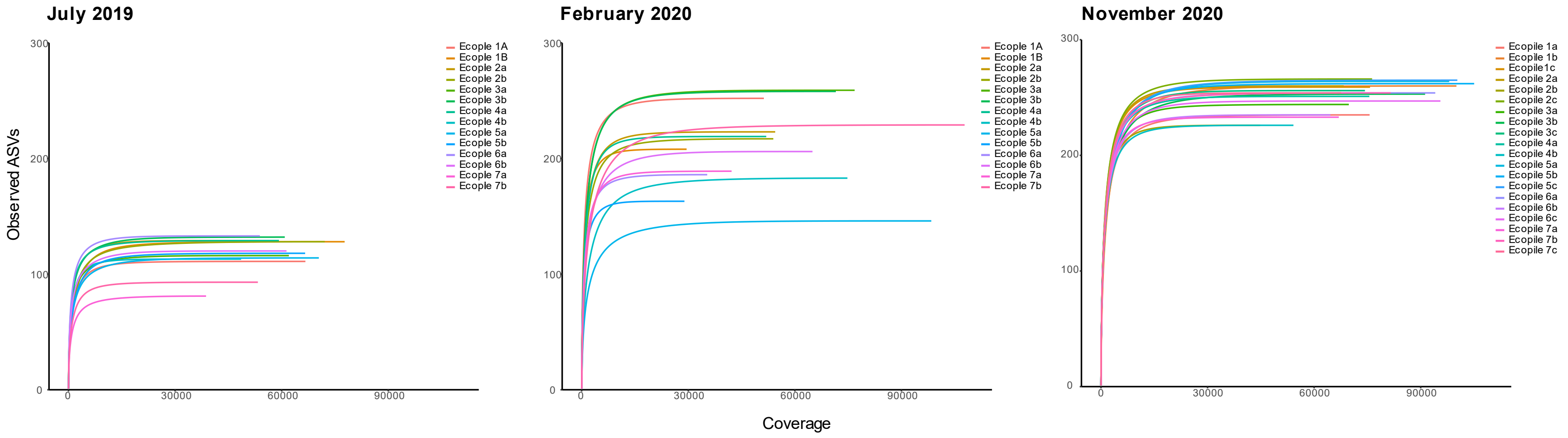

Figure S4. Fungal rarefaction curves across Ecopiles and timepoints. Rarefaction curves of 18S fungal ASVs observed over the total reads from the different Ecopiles over the three sampling times, July 2019, February 2020 and November 2020. Each curve shows the evolution for each sample replicate.
